# Complete Genome Sequence of a Bovine Viral Diarrhea Virus Strain Isolated in Southern China

**DOI:** 10.1128/genomeA.00512-14

**Published:** 2014-06-19

**Authors:** Zhixun Xie, Qing Fan, Zhiqin Xie, Jiabo Liu, Yaoshan Pang, Xianwen Deng, Liji Xie, Sisi Luo, M. I. Khan

**Affiliations:** a Guangxi Key Laboratory of Animal Vaccines and Diagnostics, Guangxi Veterinary Research Institute, Nanning, Guangxi Province, China; b Department of Pathobiology & Veterinary Science, University of Connecticut, Storrs, Connecticut, USA

## Abstract

We report here the full-length RNA genomic sequence of the bovine viral diarrhea virus (BVDV) strain GX4, isolated from a cow in southern China. Studies indicate that BVDV GX4 belongs to the BVDV-1b subtype. This report will help in understanding the epidemiology and molecular characteristics of BVDV in southern China cattle.

## GENOME ANNOUNCEMENT

Bovine viral diarrhea (BVD) has a high prevalence rate and low mortality rate, leading to significant economic losses (1). Bovine viral diarrhea virus (BVDV) is a positive-sense single-stranded RNA virus, with a genome size of approximately 12.5 kb. BVDV is a member of the genus *Pestivirus* in the family *Flaviviridae*. BVDV is classified into two biotypes, cytopathogenic and noncytopathogenic, based on the presence or absence, respectively, of cytopathogenic effects in cell cultures. In addition, there are two major genotypes, BVDV1 and BVDV2, based on genetic relatedness (2). BVDV1 has at least 15 subtypes, and the complete genomic sequences of BVDV-1a, -1b, -1d, -1e, -1m, 1k,, and BVDV-2a and -2b have been reported (3–5).

A strain of BVDV, named GX4, was isolated from a dairy farm in Guangxi Province in southern China. Fecal swabs were collected from a Holstein cow with typical acute diarrheal disease. The swabs were eluted with phosphate-buffered saline (PBS) (pH 7.2), inoculated onto MDBK monolayer cultures, and incubated at 37°C for 4 days. No cytopathic effect was observed after eight serial passages. Viral genomic RNA was extracted from GX4-infected culture supernatant using the TRIzol RNA kit (Invitrogen, USA). The Primer Premier Software was used to design 12 pairs of primers, and a BLAST search was performed to verify the oligonucleotide specificities of the primers. Reverse transcription-PCR (RT-PCR) amplifications were performed using a PrimeScript one-step RT-PCR kit (TaKaRa, Dalian, China) (6). The amplified products were purified and cloned into the pMD18-T vector (TaKaRa) and then sequenced (Invitrogen, Guangzhou, China). The sequences were assembled and manually edited to generate the final genome sequence using the DNAStar MegAlign software.

The genome of GX4 comprises 12,218 nucleotides, with a 3′ untranslated region (UTR), a 5′ UTR, and one open reading frame (ORF) encompassing 3,898 amino acids. The complete sequences and the deduced amino acid sequences of GX4 were compared with those of published BVDV sequences. The homologies at the nucleotide level are 89.0% to ~99.2%, and the homologies at the deduced amino acid level are 88.6% to ~97.2% for the BVDV-1b subtype. Immunogenicity studies have shown that the nonstructural 3 (NS3) and envelope 2 (E2) glycoproteins are crucial components for eliciting neutralizing antibodies in cattle (7, 8). However, the amino acid sequence of E2 of GX4, as with the other 29 BVDV-1b subtypes, is highly variable, whereas that of NS3 is conserved. The NS3 glycoprotein has been thought to be a good candidate for use as an immunogen in the development of subunit vaccines or diagnostic kits (9, 10).

Phylogenetic tree analysis indicated that GX4 is closely related to strain Av69 VEDEVAC (GenBank accession no. KC695814.1) from Lanzhou, China; the major difference between these strains is the presence of 45 nucleotides at positions 4307 to 4351 in Av69 VEDEVAC. However, these nucleotides are not present in BVDV-1b strains, indicating that BVDV GX4 belongs to the BVDV-1b subtype. This report will help to understand the epidemiology and molecular characteristics of BVDV in Guangxi cattle.

### Nucleotide sequence accession number.

The complete genomic sequence of BVDV GX4 has been deposited in GenBank under the accession no. KJ689448.
